# Shen Qi Wan-Containing Serum Alleviates Renal Interstitial Fibrosis via Restraining Notch1-Mediated Epithelial-Mesenchymal Transition

**DOI:** 10.1155/2023/3352353

**Published:** 2023-02-06

**Authors:** Hongshu Chen, Xiaojie Zhou, Yuanxiao Yang, Yaorong Feng

**Affiliations:** ^1^The First Affiliated Hospital of Zhejiang Chinese Medical University (Zhejiang Provincial Hospital of Chinese Medicine), Hangzhou, Zhejiang 310006, China; ^2^Academy of Chinese Medical Sciences, Zhejiang Chinese Medical University, Hangzhou, Zhejiang 310053, China; ^3^School of Basic Medical Sciences and Forensic Medicine, Hangzhou Medical College, Hangzhou, Zhejiang 310053, China; ^4^The Second Affiliated Hospital of Zhejiang Chinese Medical University, Hangzhou, Zhejiang 310005, China

## Abstract

**Objective:**

Shen Qi Wan (SQW) is the most classic prescription for the clinical therapy of chronic kidney disease in China. Nevertheless, the function of SQW in renal interstitial fibrosis (RIF) has not been clearly clarified. Our purpose was to explore the protective function of SQW on RIF.

**Methods:**

After intervention with SQW-containing serum alone at increasing concentrations (2.5, 5, and 10%) or in combination with siNotch1, the transforming growth factor-beta (TGF-*β*)-induced HK-2 cell viability, extracellular matrix (ECM)-, epithelial-mesenchymal transition (EMT), and Notch1 pathway-associated protein expressions were assessed by cell counting kit-8, qRT-PCR, western blot, and immunofluorescence assays.

**Results:**

SQW-containing serum intensified the viability of TGF-*β*-mediated HK-2 cells. Besides, it augmented the collagen II and E-cadherin levels, and weakened the fibronectin, *α*-SMA, vimentin, N-cadherin, and collagen I levels in HK-2 cells triggered by TGF-*β*. Moreover, it is found that TGF-*β* led to the upregulation of Notch1, Jag1, HEY1, HES1, and TGF-*β* in HK-2 cells, which was partially offset by SQW-containing serum. Furthermore, cotreatment of SQW-containing serum and Notch1 knockdown further apparently alleviated the Notch1, vimentin, N-cadherin, collagen I, and fibronectin levels in HK-2 cells induced by TGF-*β*.

**Conclusion:**

Collectively, these findings elucidated that SQW-containing serum attenuated RIF via restraining EMT through the repression of the Notch1 pathway.

## 1. Introduction

In recent years, with the increasing incidence of diseases such as diabetes, hypertension, and obesity, the incidence of chronic kidney disease (CKD) is also growing year-by-year [1]. The global estimated prevalence of CKD is 13.4%, which brings a huge health and economic burden to patients [2]. The mounting medical and economic burden brought by CKD has been highly concerning to the World Health Organization [3]. In the next 20 years, the impact of CKD on health will rise from 16th to 5th in the world [4]. Regardless of the cause of CKD, renal fibrosis is the ultimate coconsequence and can be hardly reversed [5]. Currently, the treatment options for CKD or renal fibrosis are limited, such as control of protein, salt, and lipid intake, control of blood pressure and blood sugar, and renin-angiotensin system (RAS) blockade, but the efficacy is not satisfactory [6]. Therefore, actively exploring the pathogenesis of CKD and developing effective antifibrotic drugs has become an urgent problem in the field of nephropathy.

Renal interstitial fibrosis (RIF) is a clinicopathological change characterized by the excessive accumulation of extracellular matrix (ECM) in the renal interstitium, destruction of the renal structure, and loss of function and is a common pathway for all CKD progression to the end-stage renal disease (ESRD) [7, 8]. The severity of RIF is a significant basis for judging the prognosis of kidney diseases [9]. Previous studies on the mechanism of RIF mainly focus on inflammation, apoptosis, proliferation, and activation of fibroblasts, as well as oxidative stress, cytokines, and the signaling cascade [10, 11]. At present, studies have clarified that epithelial-mesenchymal transition (EMT) is also involved in the pathological repair after kidney injury and is related to the progression of RIF to CKD, which is another important mechanism for the formation of RIF [12–14]. In RIF, many cytokines have been discovered to be participated in the EMT process, including transforming growth factor *β* (TGF-*β*), platelet-derived growth factor (PDGF), and connective tissue growth factor (CTGF) [15–17]. Among many EMT-inducing factors, TGF-*β*1 is considered to be one of the strongest EMT-inducing factors [18]. TGF-*β*1 can induce the transdifferentiation of myofibroblasts from different sources such as macrophages, epithelial cells, and fibroblasts by activating the classic Smad-dependent and non-Smad pathways Notch/Jagged, Wnt/*β*-catenin, and Hedgehog pathways, and promote the formation of RIF [19–22]. Exploring how to delay or even reverse RIF via the EMT process so as to better protect kidney function has always been a research hotspot.

With the development of traditional Chinese medicine (TCM), TCM compounds exhibit certain advantages in the treatment of CKD due to their multiple components, multiple targets, and low side effects [23, 24]. From the perspective of TCM, “kidney-yang deficiency” is the internal condition for the development of renal fibrosis. Early treatment of the warming kidney and benefiting qi drugs can delay the process of renal fibrosis. Shen Qi Wan (SQW) is a representative prescription for tonifying the kidney in Chinese medicine and is derived from Jingui Yaol*ü*e, written by Zhang Zhongjing. SQW is composed of *Radix Rehmanniae Recens* 24 g, *Dioscoreae Rhizoma* 12 g, *Corni Fructus* 12 g, *Alismatis Rhizoma* 9 g, *Poria* 9 g, *Moutan Cortex* 9 g, *Cinnamomi Ramulus* 3 g, and *Aconiti Lateralis Praeparata Radix* 3 g [25]. A study confirmed that the *Alismatis Rhizoma*, the component of the SQW, inhibited the TGF-*β*1 and ANG-induced expressions of collagen I, fibronectin, *α*-SMA, vimentin, and E-cadherin in cells, attenuating the process of EMT [26]. In addition, *Dioscoreae Rhizoma*, which is related to the SQW, was found to inhibit the invasion and migration of HepG2 cells by reversing TGF-*β*1-induced EMT [27]. Our previous data confirmed that SQW can evidently reduce adenine-mediated RIF, which is closely related to the EMT, and its mechanism is related to the repression of TGF-*β*1/Smad signaling [25]. Nevertheless, the mechanism by which SQW alleviates RIF is unclear.

TGF-*β*1 induces the secretion of fibrotic factors in HK-2 cells, a commonly used cell model of renal fibrosis [28]. Therefore, in this study, HK-2 cells induced by TGF-*β*1 were used as the experimental model *in vitro* to explore the effect of SQW-containing serum on the EMT, ECM, and Notch1 pathways of HK-2 cells. This study aims to clarify that SQW-containing serum may weaken EMT by interfering with the conduction of the Notch1 pathway to have a synergistic anti-RIF effect and provide a new theoretical basis for the application and research of SQW in RIF.

## 2. Materials and Methods

### 2.1. Preparation of SQW Suspension

In brief, 400 concentrated pills (each 4 pills is equivalent to 1.5 g of the original medicinal materials) were placed in a mortar to be ground into a fine powder. After dissolving with distilled water, they were placed in an ultrasonic instrument for 30 min to fully dissolve. Then, SQW was concentrated to a final concentration of 2 g/mL of the original medicinal materials, followed by storage at 4°C for later use.

### 2.2. Animal Care

Twenty 8 week-old male SD rats (200 ± 20 g) were provided by the Chinese Academy of Sciences of Shanghai Laboratory Animal Center. The animal research was ratified by the Ethics Committee of the Animal Center of Zhejiang Eyong Pharmaceutical Research and Development Center (animal use license number: SYXK (Zhe) 2021-0033). All rats were adapted for 1 week under pathogen-free conditions (20 ± 2°C, 12 h light/dark cycle) with a regular pellet diet and water.

### 2.3. Preparation of SQW-Containing Serum

Twenty rats were randomly separated into the SQW group (*n* = 10, 3 g/kg) and the control group (*n* = 10). The SQW group was given 3 g/kg SQW suspension (0.3 g/mL) by intragastric administration, twice a day, for 5 consecutive days. The control group was given 0.9% saline, twice a day, for 5 consecutive days. During the administration period, all rats ate normally and fasted 12 h before the last administration. One hour after the last administration, pentobarbital sodium (40 mg/kg intraperitoneally) was applied to anesthetize the rats. Subsequently, the blood samples were acquired through the abdominal aorta. The blood sample was allowed to stand for 60 min at 4°C, followed by centrifugation (3000 r/min for 15 min). Then, the serum was harvested and inactivated at 56°C for 0.5 h. After filtration, we got the SQW-containing serum and normal serum. All serum was put at −20°C for further *in vitro* experiments.

### 2.4. Cell Culture

Human proximal tubule epithelial cell line HK-2 (CL-0109), bought from Procell (China), was grown in the MEM complete medium (CM-0109, Procell, China). HK-2 cells were put in an incubator (37°C, 5% CO_2,_ BB150, Thermo Fisher, USA) for culture.

### 2.5. Cell Treatment

To analyze the function of SQW on HK-2 cells, cells were assigned to 5 groups. The control group was exposed to 10% normal serum for 48 h. The TGF-*β* group was intervened with 10 ng/mL TGF-*β*1 for 48 h. The concentration of TGF-*β*1 was consistent with a similar previous study [29]. The TGF-*β* + SQW-low (SL) group was stimulated with 7.5% normal serum, 2.5% SQW-containing serum, and 10 ng/mL TGF-*β*1 for 48 h. The TGF-*β* + SQW-medium (SM) group was subjected to 5% normal serum, 5% SQW-containing serum, and 10 ng/mL TGF-*β*1 for 48 h. The TGF-*β* + SQW-high (SH) group was treated with 10% SQW-containing serum and 10  ng/mL TGF-*β*1 for 48 h. Then, to further probe the impacts of Notch1 on HK-2 cells, cells were separated to the control group, TGF-*β* group, small interfering RNA targeting Notch1 (siNotch1) group (cells transfected with siNotch1 were treated with TGF-*β*1), and siNotch1 + SH group (cells transfected with siNotch1 were intervened with 10% SQW-containing serum and 10  ng/mL TGF-*β*1 for 48 h).

### 2.6. Cell Viability Experiment

Cell counting kit-8 (CCK-8, HY-K0301), supplied from MCE (USA), was earmarked for evaluating the cell viability. The logarithmic growth period of HK-2 cells (10000 cells/well) was appended to the 96-well plates and put in the incubator for 24 h. After treatment, 10 *μ*L CCK-8 solution was applied to stimulate cells for another 3 h. In the end, a microplate reader (CMaxPlus, MD, USA) was earmarked for examining the absorbance (450 nm).

### 2.7. Real-Time Quantitative PCR (qRT-PCR)

Total RNA extraction kit (R1200), bought from Solarbio (China), was applied to isolate total RNA from cells. Then, a one-stepSuperRT-PCR mix kit (T2240, Solarbio, China) was applied to conduct the qRT-PCR reaction. Afterward, the signals were examined in a PCR system (EDC-810, Eastwin Life Sciences, Inc.). GAPDH was employed as the normalization control. The relative levels of gene were counted utilizing 2^−ΔΔCT^. The primers are displayed in Table 1.

### 2.8. Western Blot

Total protein from cells was extracted with RIPA buffer (abs9229, Absin, China). The extracted protein was centrifuged and then quantified with the BCA kit (pc0020, Solarbio, China). After electrophoresis, they were electrotransferred onto the nitrocellulose membrane. Then, 5% nonfat milk was taken to block the membrane. After rinsing, the blocked membrane was subjected to primary antibodies at 4°C all night. The next day, an antirabbit secondary antibody (ab7090, Abcam, UK) was added. After reacting at 37°C for 1 h, a color reagent (1705061, BIO-RAD, USA) was taken to visualize the blots. Finally, the blots were developed in ChemiScope 3300 mini equipment (Clinx, China). The primary antibodies of collagen II (1: 1000, ab34712), fibronectin (1: 1000, ab268020), *α*-SMA (1: 50000, ab124964), Notch1 (1: 2000, ab52627), Jag1 (1: 500, ab7771), HEY1 (1: 3000, ab154077), HES1 (1: 1000, ab108937), TGF-*β* (1: 1000, ab215715), E-cadherin (1: 50000, ab40772), vimentin (1: 5000, ab92547), N-cadherin (1: 20000, ab76011), collagen I (1: 1000, ab260043), and GAPDH (ab181602) were bought from Abcam (UK).

### 2.9. Immunofluorescence

HK-2 cells (1 × 10^4^) appended to glass coverslips in 24-well plates were rinsed after treatment. Cells were fixed with 4% paraformaldehyde. After rinsing, a blocking reagent (ST025, Beyotime, China) was taken to seal cells for 1 h at 37°C. Then, cells were subjected to anti-Notch1 antibody (1: 150), anti-Jag1 antibody (1: 200), anticollagen I antibody (1: 250), anti-*α*-SMA antibody (1: 500), and antivimentin antibody (1: 1000) all night at 4°C, followed by the addition of goat antirabbit IgG H&L (Alexa Fluor® 488, 1: 1000, ab150077) or Alexa Fluor® 594 (1: 1000, ab150080) for 120 min at 37°C. Thereafter, DAPI (C1005, Beyotime, China) was employed to stain the nucleus for 5 min. Fluorescence images were acquired utilizing a fluorescence microscope (Ts2-FC, Nikon, Japan) after blocking with the antifade mounting medium (P0128S, Beyotime, China).

### 2.10. Statistics

All analyses were computed with the SPSS software (16.0, IBM, USA). The differences among multiple groups were compared by one-way ANOVA, followed by the SNK test. The Kruskal−Wallis *H* test was employed for those with uneven variance. Data were displayed as mean ± standard deviation. *P* < 0.05 was deemed statistically significant.

## 3. Results

### 3.1. SQW-Containing Serum Intensified the Viability of TGF-*β*-Mediated HK-2 Cells

**Figure 1:**
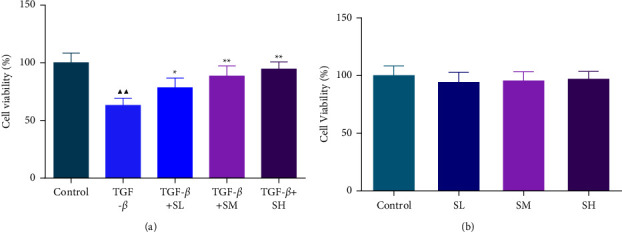
SQW-containing serum intensified the viability of TGF-*β*-mediated HK-2 cells. (a-b) The role of Shen Qi Wan (SQW)-containing serum on the viability of transforming growth factor-beta (TGF-*β*)-mediated HK-2 cells and HK-2 cells viability, which was directly influenced by the SQW-containing serum, was assessed by cell counting kit-8 (CCK-8). ^▲▲^*P* < 0.01 vs. control; ^★^*P* < 0.05 and ^★★^*P* < 0.01 vs. TGF-*β*.

In our research, we first adopted HK-2 cells treated with 10 ng/mL TGF-*β* to test the function of SQW-containing serum. CCK-8 experiment clarified that TGF-*β* led to the reduction of HK-2 cell viability (Figure 1(a)), *P* < 0.01). Importantly, the repression of TGF-*β* on HK-2 cell viability was reversed by SQW-containing serum in a concentration-dependent way (Figure 1(a)), *P* < 0.05). And different dosages of the SQW-containing serum have no impact on the HK-2 cell viability (Figure 1(b)).

### 3.2. SQW-Containing Serum Augmented the Collagen II Level and Weakened the Fibronectin and *α*-SMA Levels in HK-2 Cells Triggered by TGF-*β*

**Figure 2:**
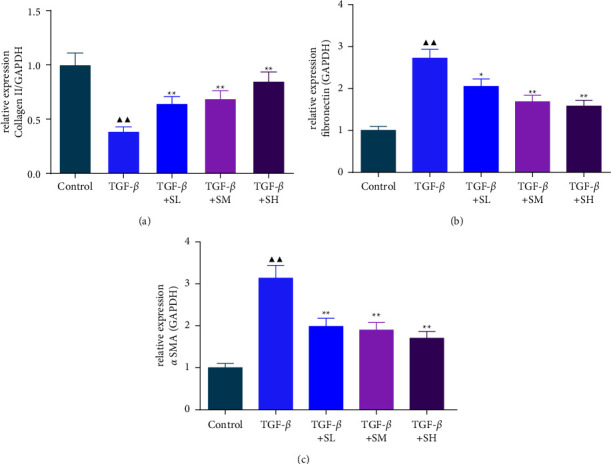
SQW-containing serum led to the increase of collagen II and the reduction of fibronectin and *α*-SMA in TGF-*β*-mediated HK-2 cells. (a–c) The role of SQW-containing serum on the collagen II, fibronectin, and *α*-SMA levels in TGF-*β*-mediated HK-2 cells was tested by qRT-PCR, with GAPDH as the endogenous control. ^▲▲^*P* < 0.01 vs. control; ^★^*P* < 0.05 and ^★★^*P* < 0.01 vs. TGF-*β*.

**Figure 3:**
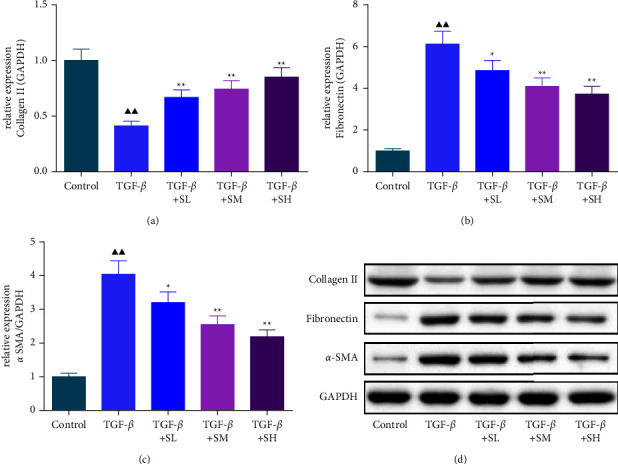
The impact of SQW-containing serum on the collagen II, fibronectin, and *α*-SMA levels in HK-2 cells induced by TGF-*β*. (a–d) The impacts of SQW-containing serum on the collagen II, fibronectin, and *α*-SMA levels in TGF-*β*-mediated HK-2 cells were tested by western blot, with GAPDH as the endogenous control. ^▲▲^*P* < 0.01 vs. control; ^★^*P* < 0.05 and ^★★^*P* < 0.01 vs. TGF-*β*.

In the next research, qRT-PCR and western blot experiments were employed to probe the mechanism behind SQW weakening EMT in HK-2 cells triggered by TGF-*β*. As exhibited in Figures 2(a)–2(c) and Figures 3(a)–3(d), TGF-*β* caused the decrease of collagen II and the elevation of fibronectin and *α*-SMA (*P* < 0.01). However, SQW-containing serum repressed TGF-*β*-triggered ECM and EMT in HK-2 cells by the reduction of *α*-SMA and fibronectin, and the promotion of collagen II (Figures 2(a)–2(c) and Figures 3(a)–3(d), *P* < 0.05).

### 3.3. SQW-Containing Serum Repressed the Notch1 Pathway, TGF-*β*, and N-Cadherin Level and Enhanced the E-Cadherin Level in HK-2 Cells Mediated by TGF-*β*

**Figure 4:**
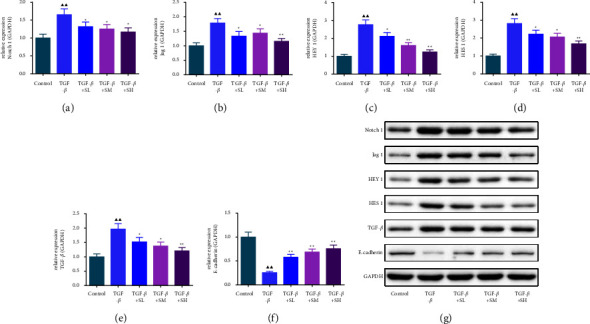
SQW-containing serum repressed the Notch1 pathway and TGF-*β* level and enhanced the E-cadherin level in TGF-*β*-mediated HK-2 cells. (a–g) The impact of SQW-containing serum on the Notch1 pathway, TGF-*β*, and E-cadherin levels was assessed by western blot, with GAPDH as the internal reference. ^▲▲^*P* < 0.01 vs. control; ^★^*P* < 0.05 and ^★★^*P* < 0.01 vs. TGF-*β*.

**Figure 5:**
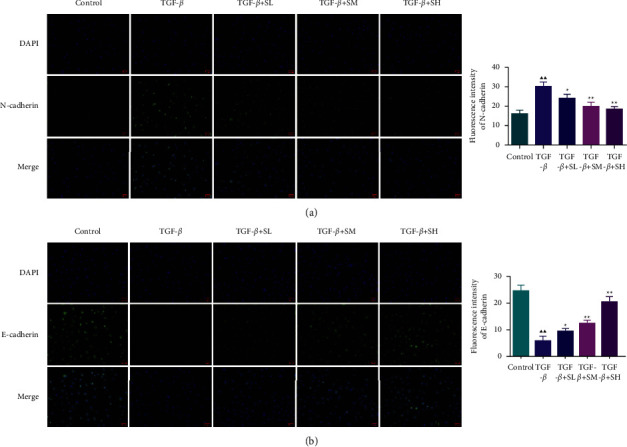
SQW-containing serum reduced the N-cadherin level and enhanced the E-cadherin level in HK-2 cells mediated by TGF-*β*. (a-b) Immunofluorescence was used to observe the expressions of the N-cadherin and E-cadherin level of HK-2 cells in each group. ^▲▲^*P* < 0.01 vs. control; ^★^*P* < 0.05 and ^★★^*P* < 0.01 vs. TGF-*β*.

**Figure 6:**
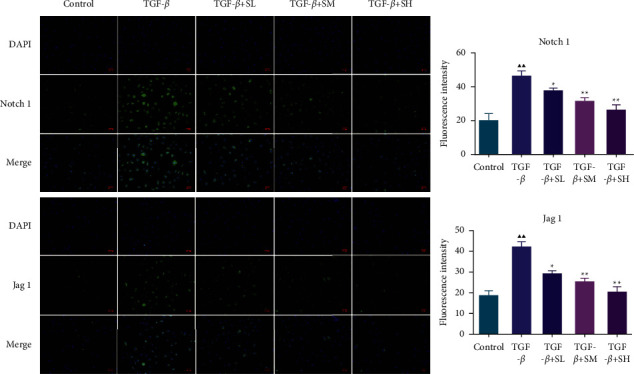
The impact of SQW-containing serum on the fluorescence intensities of Notch1 and Jag1 in HK-2 cells mediated by TGF-*β*. The impact of SQW-containing serum on the fluorescence intensities of Notch1 and Jag1 in HK-2 cells mediated by TGF-*β* was examined by immunofluorescence. ^▲▲^*P* < 0.01 vs. control; ^★^*P* < 0.05 and ^★★^*P* < 0.01 vs. TGF-*β*.

In this part, we tested the Notch1 pathway, which is tightly related to RIF. The western blot elucidated that the intervention of TGF-*β* led to the upregulation of Notch1, Jag1, HEY1, HES1, and TGF-*β* and the downregulation of E-cadherin in HK-2 cells, which was partially offset by SQW-containing serum (Figures 4(a)–4(g), *P* < 0.05). Besides, we used immunofluorescence assay to assess the fluorescence intensity of the N-cadherin and the E-cadherin, the results reconfirmed that SQW-containing serum elevated the expression of E-cadherin while reduced the N-cadherin in HK-2 cells mediated by TGF-*β* (Figure 5(a)–5(b), *P* < 0.05 or *P* < 0.01). Simultaneously, we also assessed the fluorescence intensity of Notch1 and Jag1. We discovered that SQW-containing serum weakened the fluorescence intensities of Notch1 and Jag1 in HK-2 cells mediated by TGF-*β* (Figure 6, *P* < 0.05). Since the high-dose group of SQW-containing serum has the most obvious protective effect on RIF, we chose 10% SQW-containing serum for follow-up experiments.

### 3.4. The Impacts of SQW-Containing Serum and siNotch1 on EMT-Related Factors in HK-2 Cells Mediated by TGF-*β*

**Figure 7:**
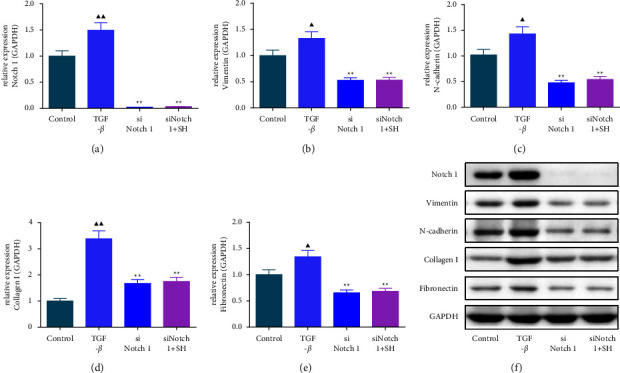
The impacts of SQW-containing serum and siNotch1 on EMT-related factors in HK-2 cells mediated by TGF-*β*. (a–f) The impacts of SQW-containing serum and small interference RNA Notch1 (siNotch1) on EMT-related factors in HK-2 cells mediated by TGF-*β* were assessed by western blot, with GAPDH as the housekeeping reference. ^▲^*P* < 0.05 and ^▲▲^*P* < 0.01 vs. control; ^★★^*P* < 0.01 vs. TGF-*β*.

**Figure 8:**
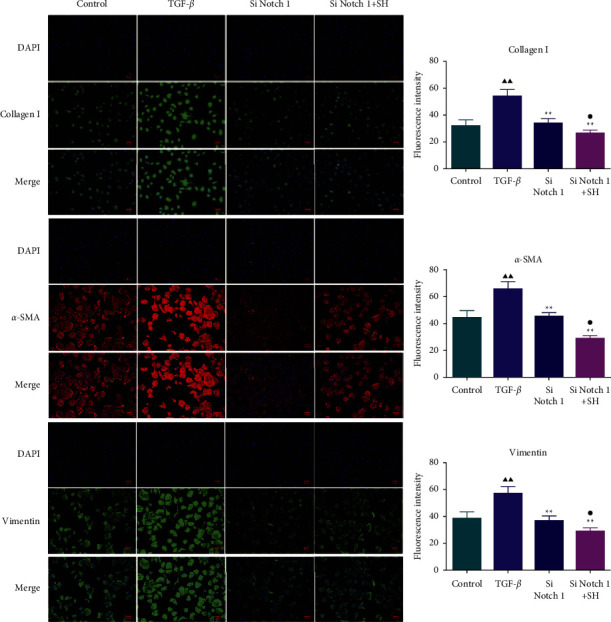
The roles of SQW-containing serum and siNotch1 on the fluorescence intensities of collagen I, *α*-SMA, and vimentin in TGF-*β*-mediated HK-2 cells. The roles of SQW-containing serum and siNotch1 on the fluorescence intensities of collagen I, *α*-SMA, and vimentin in TGF-*β*-mediated HK-2 cells were tested by immunofluorescence. ^▲▲^*P* < 0.01 vs. control; and ^★★^*P* < 0.01 vs. TGF-*β*; ^●^*P* < 0.05 vs. siNotch1 group.

To further confirm that the protective effect of SQW-containing serum on RIF was correlated with the repression of the Notch1 pathway, HK-2 cells transfected with Notch1 silencing were intervened with 10% SQW-containing serum and 10 ng/mL TGF-*β* for 48 h. Notch1 silencing alone or combined with SQW-containing serum strongly alleviated the Notch1, vimentin, N-cadherin, collagen I, and fibronectin levels in HK-2 cells induced by TGF-*β* (Figures 7(a)–7(f), *P* < 0.05). We also demonstrated by the immunofluorescence assay that cotreatment of SQW-containing serum and siNotch1 apparently repressed the fluorescence intensities of collagen I, *α*-SMA, and vimentin in TGF-*β*-mediated HK-2 cells compared to the siNotch group (Figure 8, *P* < 0.05).

## 4. Discussion

RIF and the degree of the renal tubular atrophy are significant factors determining the severity of different forms of renal diseases. Renal tubular epithelial cells (RTECs) are the major targets of renal interstitial damage [30]. Under the stimulation of continuous ischemia and hypoxia, proteinuria, and a variety of profibrotic factors, RTECs secrete multiple chemokines, inflammatory factors, and vasoactive factors into the renal interstitium in order to adapt to the changes in the microenvironment so as to promote the inflammatory response and fibrosis process of the renal interstitium [14]. TGF-*β*1 is considered to be a major agonist in the formation of renal fibrosis [31]. Meanwhile, HK-2 itself is also a target of TGF-*β*1. HK-2 can transform into cells with obvious myofibroblast morphology under the induction of TGF-*β*1, such as changes in cell polarity, actin filaments, and dense bodies, resulting in excessive deposition of ECM in the renal interstitium and formation of RIF [32, 33]. Based on the important role of HK-2 cells and its correlation with TGF-*β*1, this study stimulated HK-2 cells with TGF-*β*1 to mimic RIF *in vitro*, and investigated the anti-RIF effect of SQW-containing serum. And this study demonstrated that SQW can attenuate the TGF-*β*-mediated EMT process in HK-2 cells to alleviate RIF *in vitro*.

It was reported that during renal fibrosis, RTECs can differentiate into mesenchymal cells and transform into fibroblasts, becoming a considerable source of ECM [34]. The transformation of epithelial cells to mesenchymal cells is characterized by the absence of epithelial marker E-cadherin and the increase of mesenchymal marker *α*-SMA, N-cadherin, vimentin, and fibronectin [35, 36]. Fibronectin is one of the main components of the ECM, which can be formed in the early stage of fibrosis and is one of the intuitive indicators of renal fibrosis [37]. N-cadherin is a cadherin expressed in proximal tubules and generates a pivotal function in maintaining the integrity and polarity of RTECs [38]. Under normal circumstances, there is basically no expression of *α*-SMA in the glomerulus, renal tubules, and renal interstitium, and *α*-SMA is considered as one of the symbols of EMT in TECs [39]. According to Lu et al.'s research, it is proved that with the induction of the TGF-*β*1, it notably augmented the expression of *α*-SMA, but ameliorated the expression of E-cadherin, indicating the completion of transformation from epithelial cells to mesenchymal cells in Madin−Darby canine kidney cells [40]. Consistently, we uncovered that TGF-*β* led to the downregulation of collagen II and E-cadherin and the upregulation of fibronectin, *α*-SMA, vimentin, N-cadherin, and collagen I in HK-2 cells. Therefore, inhibition of EMT and ECM can be an effective way to improve RIF.

At present, TCM has been widely utilized as an alternative therapy for kidney diseases. For instance, some scholars clarified that the *Huangkui* capsule mitigated renal tubular EMT in diabetic nephropathy in rats through the repression of the NLRP3 inflammasome and TLR4/NF-*κ*B pathway [41]. In TGF-*β*1-inducedHK-2 cells, ganoderic acid weakened the ECM and EMT through the inhibition of the TGF-*β*/Smad and MAPK pathways [42]. Shan et al. illuminated that *Astragalus membranaceus* improved RIF via impeding tubular EMT *in vivo* and *in vitro* [43]. In our study, we proved the role of SQW in TGF-*β*-mediated HK-2 cells for the first time. We discovered that SQW-containing serum weakened the expressions of fibronectin and collagen I, and this function was accompanied by the suppression of EMT process by restraining *α*-SMA, vimentin, and N-cadherin levels and elevating the E-cadherin level in HK-2 cells triggered by TGF-*β*, unveiling that SQW may exhibit an anti-RIF effect by repressing ECM and EMT processes.

It was reported that Notch is also a considerable modulatory pathway for EMT and renal fibrosis [44]. Notch pathway is low or not expressed in the adult normal kidney tissue, but is reactivated in various kidney diseases [45]. Expression of the Notch receptor in RTECs cultured *in vitro* can drive epithelial cells to develop EMT [46]. In addition, the increase of Notch expression in the tubule epithelium is associated with the continuous increase of TGF-*β*1 [47]. Inhibition of the Notch1 pathway can alleviate EMT induced by TGF-*β*1 and ameliorate RIF [48]. Some scholars found that the expressions of Notch1 and Jagged1 were significantly upregulated in the kidney tissues of IgA nephropathy, and Notch1, HEY1, and HES1 were significantly reduced after administration of the Notch1 pathway inhibitor DAPT, thereby alleviating RIF [49]. Notch pathway inhibitors were found to weaken glomerulosclerosis and RIF by decreasing the expression of Jagged1, Notch1, NICD1, HEY1, HES1*α*-SMA, and fibronectin in uremic rats [50]. Thus, inhibition of the Notch pathway can alleviate RIF to a certain extent. In this research, our results uncovered that TGF-*β* led to the upregulation of Notch1, Jag1, HEY1, HES1, and TGF-*β* in HK-2 cells, which was partially offset by SQW-containing serum. To further explore whether the Notch1 pathway affects the effect of SQW on RIF, Notch1 silence was transfected into HK-2 cells. We confirmed that Notch1 silencing or cotreatment of SQW-containing serum with Notch1 silencing mitigated ECM and EMT induced by TGF-*β*1 and improved RIF via weakening the Notch1 pathway.

In short, this study demonstrated that SQW attenuated the TGF-*β*-mediated EMT process in HK-2 cells, and its possible mechanism is associated with the inhibition of the Notch1 pathway. However, there are some shortcomings of the study, the overexpression of Notch 1 should be conducted to make the results more convincing, and there is a need to verify the mechanism in an animal model. Other potential mechanisms should be explored in subsequent studies to confirm the exact mechanism of SQW anti-RIF.

## Figures and Tables

**Table 1:** Gene sequence primers.

Gene	Forward primer	Reverse primer
Human collagen II	GTAGAGACCCGGACCCGC	ACTCTCCGAAGGGGATCTCA
Human fibronectin	ACAAGCATGTCTCTCTGCCA	TTTGCATCTTGGTTGGCTGC
Human *α*-SMA	CAGGAGGATTCCGTGCTGTT	GCTGGGTAAATGCAACCGTC
Human GAPDH	AATGGGCAGCCGTTAGGAAA	GCGCCCAATACGACCAAATC

## Data Availability

The data used to support the findings of this study are included within this article.

## References

[1] Lv J. C., Zhang L. X. (2019). Prevalence and disease burden of chronic kidney disease. *Advances in Experimental Medicine and Biology*.

[2] Lv J., Zhang L. J. A. (2019). Prevalence and disease burden of chronic kidney disease. *Advances in Experimental Medicine and Biology*.

[3] Webster A. C., Nagler E. V., Morton R. L., Masson P. (2017). Chronic kidney disease. *The Lancet*.

[4] Foreman K. J., Marquez N., Dolgert A., Fukutaki K., Fullman N., McGaughey M. (2018). Forecasting life expectancy, years of life lost, and all-cause and cause-specific mortality for 250 causes of death: reference and alternative scenarios for 2016-40 for 195 countries and territories. *Lancet (London, England)*.

[5] Xi Y., Lu X., Zhu L. (2020). Clinical trial for conventional medicine integrated with traditional Chinese medicine (TCM) in the treatment of patients with chronic kidney disease. *Medicine*.

[6] Liu X., Huang S., Wang F. (2019). Huangqi-danshen decoction ameliorates adenine-induced chronic kidney disease by modulating mitochondrial dynamics. *Evidence-based complementary and alternative medicine: eCAM*.

[7] Tang J., Jiang X., Zhou Y., Xia B., Dai Y. (2015). Increased adenosine levels contribute to ischemic kidney fibrosis in the unilateral ureteral obstruction model. *Experimental and Therapeutic Medicine*.

[8] Djudjaj S., Boor P. (2019). Cellular and molecular mechanisms of kidney fibrosis. *Molecular Aspects of Medicine*.

[9] Lee S. Y., Kim S. I., Choi M. E. (2015). Therapeutic targets for treating fibrotic kidney diseases. *Translational Research: The Journal of Laboratory and Clinical Medicine*.

[10] Liu B. C., Tang T. T., Lv L. L. (2019). How tubular epithelial cell injury contributes to renal fibrosis. *Advances in Experimental Medicine and Biology*.

[11] Humphreys B. D. (2018). Mechanisms of renal fibrosis. *Annual Review of Physiology*.

[12] Xie Y., Lan F., Zhao J., Shi W. (2020). Hirudin improves renal interstitial fibrosis by reducing renal tubule injury and inflammation in unilateral ureteral obstruction (UUO) mice. *International Immunopharmacology*.

[13] Wang Z., Zhang B., Chen Z. (2020). The long noncoding RNA myocardial infarction-associated transcript modulates the epithelial-mesenchymal transition in renal interstitial fibrosis. *Life Sciences*.

[14] Lovisa S., Zeisberg M., Kalluri R. (2016). Partial epithelial-to-mesenchymal transition and other new mechanisms of kidney fibrosis. *Trends in Endocrinology and Metabolism: Trends in Endocrinology and Metabolism*.

[15] Grande M. T., Sánchez-Laorden B., López-Blau C. (2015). Snail1-induced partial epithelial-to-mesenchymal transition drives renal fibrosis in mice and can be targeted to reverse established disease. *Nature Medicine*.

[16] Lopes T. G., de Souza M. L., da Silva V. D., Dos Santos M., da Silva W. I. C., Itaquy T. P. (2019). Markers of renal fibrosis: how do they correlate with podocyte damage in glomerular diseases?. *PLoS One*.

[17] Lv W., Booz G. W., Wang Y., Fan F., Roman R. J. (2018). Inflammation and renal fibrosis: recent developments on key signaling molecules as potential therapeutic targets. *European Journal of Pharmacology*.

[18] Isaka Y. (2018). Targeting TGF-*β* signaling in kidney fibrosis. *International Journal of Molecular Sciences*.

[19] Meng X. M., Wang S., Huang X. R. (2016). Inflammatory macrophages can transdifferentiate into myofibroblasts during renal fibrosis. *Cell Death & Disease*.

[20] Higgins S. P., Tang Y., Higgins C. E., Mian B., Zhang W., Czekay R. P. (2018). TGF-*β*1/p53 signaling in renal fibrogenesis. *Cellular Signalling*.

[21] Higgins C. E., Tang J., Mian B. M., Higgins S. P., Gifford C. C., Conti D. J. (2019). TGF-*β*1-p53 cooperativity regulates a profibrotic genomic program in the kidney: molecular mechanisms and clinical implications. *Official Publication of the Federation of American Societies for Experimental Biology*.

[22] Edeling M., Ragi G., Huang S., Pavenstädt H., Susztak K. (2016). Developmental signalling pathways in renal fibrosis: the roles of Notch, Wnt and Hedgehog. *Nature Reviews Nephrology*.

[23] Zhou P., Zhang X., Guo M. (2019). Ginsenoside Rb1 ameliorates CKD-associated vascular calcification by inhibiting the Wnt/*β*-catenin pathway. *Journal of Cellular and Molecular Medicine*.

[24] Shen Y. L., Wang S. J., Rahman K., Zhang L. J., Zhang H. (2018). Chinese herbal formulas and renal fibrosis: an overview. *Current Pharmaceutical Design*.

[25] Chen H., Xu Y., Yang Y., Zhou X., Dai S., Li C. (2017). Shenqiwan ameliorates renal fibrosis in rats by inhibiting TGF-*β*1/smads signaling pathway. *Evidence-based Complementary and Alternative Medicine: eCAM*.

[26] Chen H., Yang T., Wang M. C., Chen D. Q., Yang Y., Zhao Y. Y. (2018). Novel RAS inhibitor 25-O-methylalisol F attenuates epithelial-to-mesenchymal transition and tubulo-interstitial fibrosis by selectively inhibiting TGF-*β*-mediated Smad3 phosphorylation. *Phytomedicine: International Journal of Phytotherapy and Phytopharmacology*.

[27] Chen B., Zhou S., Zhan Y. (2019). Dioscin inhibits the invasion and migration of hepatocellular carcinoma HepG2 cells by reversing TGF-*β*1-inducedepithelial-mesenchymal transition. *Molecules*.

[28] Sun Z., Ma Y., Chen F., Wang S., Chen B., Shi J. (2018). miR-133b and miR-199b knockdown attenuate TGF-*β*1-induced epithelial to mesenchymal transition and renal fibrosis by targeting SIRT1 in diabetic nephropathy. *European Journal of Pharmacology*.

[29] Shen H., He Q., Dong Y., Shao L., Liu Y., Gong J. (2020). Upregulation of miRNA-1228-3p alleviates TGF-*β*-induced fibrosis in renal tubular epithelial cells. *Histology & Histopathology*.

[30] Zhou T., Luo M., Cai W. (2018). Runt-related transcription factor 1 (RUNX1) promotes TGF-*β*-induced renal tubular epithelial-to-mesenchymal transition (EMT) and renal fibrosis through the PI3K subunit p110*δ*. *EBioMedicine*.

[31] Madne T. H., Dockrell M. E. C. (2018). TGF*β*1-mediated expression and alternative splicing of Fibronectin Extra Domain A in human podocyte culture. *Cellular and Molecular Biology*.

[32] Boor P., Ostendorf T., Floege J. (2010). Renal fibrosis: novel insights into mechanisms and therapeutic targets. *Nature Reviews Nephrology*.

[33] Böttinger E. P. (2007). TGF-beta in renal injury and disease. *Seminars in Nephrology*.

[34] Sun Y. B., Qu X., Caruana G., Li J. (2016). The origin of renal fibroblasts/myofibroblasts and the signals that trigger fibrosis. *Differentiation; research in biological diversity*.

[35] Seccia T. M., Caroccia B., Piazza M., Rossi G. P. (2019). The key role of epithelial to mesenchymal transition (EMT) in hypertensive kidney disease. *International Journal of Molecular Sciences*.

[36] Thiery J. P., Acloque H., Huang R. Y., Nieto M. A. (2009). Epithelial-mesenchymal transitions in development and disease. *Cell*.

[37] Pan Z., Yang K., Wang H. (2020). MFAP4 deficiency alleviates renal fibrosis through inhibition of NF-*κ*B and TGF-*β*/Smad signaling pathways. *Official Publication of the Federation of American Societies for Experimental Biology*.

[38] Gong L., Jiang L., Qin Y., Jiang X., Song K., Yu X. (2018). Protective effect of retinoic acid receptor *α* on hypoxia-induced epithelial to mesenchymal transition of renal tubular epithelial cells associated with TGF-*β*/MMP-9 pathway. *Cell Biology International*.

[39] Chebotareva N. V., Bobkova I. N., Varshavskiĭ V. A., Golitsyna E. P., Kozlovskaia L. V. (2006). The role of smooth muscle alpha-actin in development of renal fibrosis in patients with chronic glomerulonephritis. *Terapevticheskii Arkhiv*.

[40] Lu L., Zhu J., Zhang Y., Wang Y., Zhang S., Xia A. (2019). Febuxostat inhibits TGF-*β*1-inducedepithelial-mesenchymal transition via downregulation of USAG-1 expression in Madin-Darby canine kidney cells in vitro. *Molecular Medicine Reports*.

[41] Han W., Ma Q., Liu Y. (2019). Huangkui capsule alleviates renal tubular epithelial-mesenchymal transition in diabetic nephropathy via inhibiting NLRP3 inflammasome activation and TLR4/NF-*κ*B signaling. *Phytomedicine International Journal of Phytotherapy and Phytopharmacology*.

[42] Geng X. Q., Ma A., He J. Z. (2020). Ganoderic acid hinders renal fibrosis via suppressing the TGF-*β*/Smad and MAPK signaling pathways. *Acta Pharmacologica Sinica*.

[43] Shan G., Zhou X. J., Xia Y., Qian H. J. (2016). Astragalus membranaceus ameliorates renal interstitial fibrosis by inhibiting tubular epithelial-mesenchymal transition in vivo and in vitro. *Experimental and Therapeutic Medicine*.

[44] Bielesz B., Sirin Y., Si H. (2010). Epithelial Notch signaling regulates interstitial fibrosis development in the kidneys of mice and humans. *Journal of Clinical Investigation*.

[45] Marquez-Exposito L., Cantero-Navarro E., Lavoz C. (2018). Could Notch signaling pathway be a potential therapeutic option in renal diseases?. *Nefrologia*.

[46] Du R., Sun W., Xia L., Zhao A., Yu Y., Zhao L. (2012). Hypoxia-induceddown-regulation of microRNA-34a promotes EMT by targeting the Notch signaling pathway in tubular epithelial cells. *PLoS One*.

[47] Xiao Z., Zhang J., Peng X., Dong Y., Jia L., Li H. (2014). The Notch *γ*-secretase inhibitor ameliorates kidney fibrosis via inhibition of TGF-*β*/Smad2/3 signaling pathway activation. *The International Journal of Biochemistry & Cell Biology*.

[48] Zhou H., Gao L., Yu Z. H., Hong S. J., Zhang Z. W., Qiu Z. Z. (2019). LncRNA HOTAIR promotes renal interstitial fibrosis by regulating Notch1 pathway via the modulation of miR-124. *Nephrology*.

[49] Sheng X., Zuo X., Liu X., Zhou Y., Sun X. (2018). Crosstalk between TLR4 and Notch1 signaling in the IgA nephropathy during inflammatory response. *International Urology and Nephrology*.

[50] Chen W., Wang Z. K., Ren Y. Q., Zhang L., Sun L. N., Man Y. L. (2020). Silencing of keratin 1 inactivates the Notch signaling pathway to inhibit renal interstitial fibrosis and glomerular sclerosis in uremia. *Journal of Cellular Physiology*.

